# LUMOS - A Sensitive and Reliable Optode System for Measuring Dissolved Oxygen in the Nanomolar Range

**DOI:** 10.1371/journal.pone.0128125

**Published:** 2015-06-01

**Authors:** Philipp Lehner, Christoph Larndorfer, Emilio Garcia-Robledo, Morten Larsen, Sergey M. Borisov, Niels-Peter Revsbech, Ronnie N. Glud, Donald E. Canfield, Ingo Klimant

**Affiliations:** 1 Institute Of Analytical Chemistry And Food Chemistry, Graz University Of Technology, NAWI Graz, Stremayrgasse 9, 8010 Graz, Austria; 2 Department Of Biosciences, Aarhus University, 8000 Aarhus, Denmark; 3 Nordic Center For Earth Evolution, University Of Southern Denmark, 5230 Odense M, Denmark; CNR, ITALY

## Abstract

Most commercially available optical oxygen sensors target the measuring range of 300 to 2 μmol L^-1^. However these are not suitable for investigating the nanomolar range which is relevant for many important environmental situations. We therefore developed a miniaturized phase fluorimeter based measurement system called the LUMOS (Luminescence Measuring Oxygen Sensor). It consists of a readout device and specialized “sensing chemistry” that relies on commercially available components. The sensor material is based on palladium(II)-5,10,15,20-tetrakis-(2,3,4,5,6-pentafluorphenyl)-porphyrin embedded in a Hyflon AD 60 polymer matrix and has a K_SV_ of 6.25 x 10^-3^ ppmv^-1^. The applicable measurement range is from 1000 nM down to a detection limit of 0.5 nM. A second sensor material based on the platinum(II) analogue of the porphyrin is spectrally compatible with the readout device and has a measurement range of 20 μM down to 10 nM. The LUMOS device is a dedicated system optimized for a high signal to noise ratio, but in principle any phase flourimeter can be adapted to act as a readout device for the highly sensitive and robust sensing chemistry. Vise versa, the LUMOS fluorimeter can be used for read out of less sensitive optical oxygen sensors based on the same or similar indicator dyes, for example for monitoring oxygen at physiological conditions. The presented sensor system exhibits lower noise, higher resolution and higher sensitivity than the electrochemical STOX sensor previously used to measure nanomolar oxygen concentrations. Oxygen contamination in common sample containers has been investigated and microbial or enzymatic oxygen consumption at nanomolar concentrations is presented.

## Introduction

Online determination of oxygen concentration is extensively applied in many areas of science and technology[1–3], as oxygen is an important oxidant in both chemical and biological reactions. Monitoring oxygen concentration in the micromolar range is well established and a multitude of commercial sensor systems, both electrochemical and optical, exist. There are, however, numerous applications where lower concentrations of oxygen need to be monitored, such as in industrial process control[4], and corrosion protection[5]. Also, a significant number of aerobic or facultative anaerobic organisms grow in conditions with oxygen concentrations significantly below 1 μM (DO) [6–9]. An area of research where low-concentration oxygen monitoring is especially relevant is the investigation of oceanic oxygen-minimum zones (OMZ) containing huge volumes of anoxic or near-anoxic water[10,11], such as the eastern tropical north pacific OMZ[12]. The range of sensor systems that can determine oxygen concentrations in the nano molar range are currently very limited. One such sensor, the electrochemical STOX sensor[13], can be applied both in the laboratory and for in-situ investigations and can reliably measure concentrations down to about 10 nM. Its sensitivity compares favorably to other commercially available in-situ devices such as the Seabird and Aanderraa dissolved oxygen sensors, but it is difficult to make and is very fragile, which limits its availability and applicability—especially in laboratory setups requiring multiple sensors. A viable alternative to such electrochemical sensors are luminescence-based optical sensors[14–16]. They work by detecting a change in luminescence lifetime or intensity that is caused through physical quenching of a probe by molecular oxygen. These sensors have the benefit that they do not consume the analyte (oxygen). In addition they can be miniaturized and can be applied „contact-free“, i.e., the measurement can be performed through an optical window. The contact-free read-out is an important characteristic when working with low oxygen concentrations as the 21% oxygen in the atmosphere easily contaminates the sample by the insertion of electrochemical sensors.

We here present the LUMOS (short for Luminescence Measuring Oxygen Sensor). It is a measurement system that combines a robust sensing chemistry of very high sensitivity with a miniaturized phase fluorometry readout device. We furthermore demonstrate how the LUMOS can be used to monitor trace oxygen dynamics in various laboratory settings.

## Materials and Methods

### Materials

Platinum(II)-5,10,15,20-tetrakis-(2,3,4,5,6-pentafluorphenyl)-porphyrin (Pt-TFPP) and palladium(II)-5,10,15,20-tetrakis-(2,3,4,5,6-pentafluorphenyl)-porphyrin (Pd-TFPP) were obtained from Frontier Scientific (UK, frontiersci.com). Hyflon AD 60 and polystyrene were from Solvay (USA, solvay-plastics.com) and Fisher Scientific, respectively. Poly(phenylsilsesquioxane) 3.5–6.5% OH (PPSQ) and octafluorotoluol were acquired from ABCR (Germany, abcr.de). Other required solvents were purchased at VWR (Austria, at.vwr.com). Circular 8 mm diameter, 1 mm thick sand blasted glass substrates were custom ordered from Hilgenberg (Germany, hilgenberg-gmbh.de), aluminum casings were fabricated in house and anodized by Heuberger Eloxal (Austria, heuberger.at). DT Blue interference and RG 630 color filters were obtained from Qioptiq (Germany, qioptiq.de). Bright Red foil filters were from LEE Filters (UK, leefilters.com) 405 nm UV LEDs (Bivar) and ultrabright red LEDs were obtained from RS (Austria, at.rs-online.com) and LumiTronix (Germany, leds.de), respectively. Glucose oxidase (GOx) from *Aspergillus niger*, Tris Buffer and HgCl_2_ were obtained from Sigma-Aldrich (Swiss, sigmaaldrich.com), α-D-glucose was from Roth Chemicals (Austria, carlroth.com).

### Readout Device

The LUMOS readout device is optimized for package size and signal to noise ratio (SNR). The electronic and optical components are contained in a 33 mm diameter cylindrical anodized aluminum casing that is 24 mm high and features a mini USB plug for easy connection to a PC using the USB 2.0 standard. The LUMOS is, in essence, a miniaturized phase fluorimeter with optical components specifically tuned to maximize SNR for the selection of sensing materials it is used in combination with. It generally consists of an excitation circuit that can emit modulated light, an optics block that delivers light to and from the external sensor material, a photodetector that converts the sensor response into an output signal, and a digital signal processor (DSP) that performs the calculations, performs the signal conversion from analog to digital (and vice-versa) and handles the measurement execution and timing. This DSP communicates via a universal asynchronous receiver transmitter interface (UART) to a separate printed circuit board (PCB), which then handles the USB communication protocol (with the industry standard USB controller FT232) to the PC, with compatible software. Any alternative readout device that can be tuned to the spectral properties of the dyes and is capable of resolving lifetimes in the range of microseconds to a few milliseconds is suitable to be used in combination with the presented sensor chemistry.

### Sensor Substrate spray coating

Substrates for the LUMOS sensing sheets consist of 8 mm diameter, 1 mm thick glass disks that are sand blasted on one side to facilitate adhesion of the sensing chemistry. For best results and uniformity the sensing layers were spray-coated onto the substrates using a custom build device. The spray-coating device consists of an industrial grade airbrush from Efbe Airbrush (Germany, efbe-airbrush.de) fixed to an x-y-z table actuated by stepper motors from Isel (Germany, isel-gmbh.com). The motors are controlled by a Triple Beast Driver from Benezan Electronics (Germany, benezan-electronics.de), which also drives a fourth axis extension for the airbrush needle valve and a solenoid valve by SMC(USA, smcusa.com) for airflow switching. The spraying procedure was automated using G-Code and LinuxCNC (linuxcnc.org).

For spraying the respective dye (Pd-TFPP for trace sensors, Pt-TFPP for high range sensors, 0.01% (w/w) with respect to the solvent) and Hyflon AD 60 (2% (w/w) with respect to the solvent) were dissolved in octafluorotoluene and filled into the airbrush reservoir. The Airbrush was then led over each substrate in spirals consisting of 6 half circles with 5 repetitions. These settings were used to achieve adequate thickness of the sensing layers which was verified via measuring the signal intensity with the readout device.

### Other possible methods of sensor preparation

It is paramount for sensor performance that the sensing layers are on top of a glass substrate, as polymer substrates can act as a reservoir for oxygen and therefore influence results. In our case a large quantity of individual sensor spots were created and spray-coating proved to be the best option. For smaller batches of spots, however, knife-coating yields very good results as well. For knife-coating the respective dye (0.025% (w/w) with respect to the solvent) was dissolved along with Hyflon AD 60 (5% (w/w) with respect to the solvent) in octafluorotoluene. The resulting”cocktail” was then coated on a glass substrate, for example microscopy glass slides. For best homogeneity and adhesion the substrate should be treated with Aphaphobe CF or a similar hydrophobizing agent (a perfluorinated chlorosilane) prior to the coating. The ideal film-thickness depends on the used readout device, but using a 175 μm spacer yields good results for most setups. The sensing sheets need to be dried at 60°C for at least an hour after coating.

### Glucose oxidase (GOx) oxygen consumption experiments

A solution of 1 g of glucose and 2 g TRIS buffer in 500 ml distilled H_2_O was adjusted with 0.1 M HCl to pH 8. At the beginning of the experiment 0.5 mg GOx in 1 ml distilled H_2_O was added and the reaction was performed in a closed and stirred glass vessel where the oxygen consumption was monitored with a LUMOS device fixed to the side.

### Respiration experiments

Laboratory incubations were performed in custom-made 1.1 L glass bottles described in Tiano et al.[17]. The bottles were equipped with a port for the insertion of a STOX sensor[13] and a long pressure compensation tube. In addition, a flat circular glass window (4 cm of diameter) was built in the bottle wall in order to attach the sensor disk in the inner part and to hold the LUMOS device more easily in the outer part of the bottle.

Two respiratory experiments were conducted, using tap-water from Aarhus (Denmark) and water from the Eastern Tropical North Pacific (ETNP) OMZ. Experiments in the OMZ were performed during the Oxygen Minimum Zone Microbial Biogeochemistry Expedition 2 (OMZoMBiE2) cruise (*R/V New Horizon*; cruise NH1410; May 10-June 8, 2014). Here, water samples from 100 m depth were collected with a rosette of Niskin bottles at cruise station 16 (N 20.05734, W 107.04884; approx. 160 km offshore Manzanillo, Mexico), where the in situ oxygen concentration was below 20 nM as deduced from in situ STOX measurements[18].

To sample the water from the Danish locations, used in this study, no special permission was required. To navigate, sample and work in Mexican waters during the OMZoMBiE2 cruise aboard of R/V New Horizon, permission was requested and obtained from the Mexican government. For all stations and all experiments no endangered or protected species were involved.

Water samples for both experiments were collected in a 20 L glass bottle and treated as follows. The oxygen concentration in the water samples was first reduced to approximately 100 nM by vigorously bubbling with N_2_ gas for 15–20 minutes. After this period, samples were transferred to the incubation bottles under a continuous flow of N_2_ gas to avoid oxygen contamination. A glass-coated magnet inside the bottle driven by external magnetic stirrer ensured the homogenization of the sample during the incubation. Samples were maintained at controlled temperatures of 20°C and 14°C for the tap-water and OMZ incubations, respectively. Oxygen concentration was simultaneously monitored during the incubation by STOX sensors as described previously[12,17] and by LUMOS sensors. For demonstration purposes, the oxygen consumption of the applied tap-water was stimulated by the injection of 0.1 mL of sieved (20 μm) liquefied cattle manure, thereby adding both organic matter and bacteria. Oxygen consumption rates were calculated by simple linear regression of oxygen concentration evolution with time when the concentrations were above the oxygen limiting conditions. The continuous acquisition of data for oxygen allowed the direct fitting of an integrated Michaelis Menten kinetic as described previously[17].

### Oxygen intrusion experiments

To test for the intrusion of O_2_ into standard laboratory incubations, we performed a series of tests applying the LUMOS in combination with the trace range sensors (Pd-TFPP in Hyflon) described above. For experiments that mention headspace treatment a 2 ml or 20 ml headspace (He) was introduced into Exetainer vials (Labco, UK) or serum bottles, respectively and the vials/bottles were shaken for 15 seconds. After this the headspace was flushed and the procedure was repeated two more times. Twelve ml Exetainer vials were filled with degassed (He) MilliQ water spiked with 0.1% (v/v) saturated HgCl_2_ solution. The Exetainer vials were then closed with caps containing a butyl-rubber septum. These were either untreated caps or „deoxygenated”caps that had been kept under helium atmosphere for one month prior to use. The serum bottle experiments were performed in 120 ml serum bottles which were filled with the same degassed water solution and applying stoppers that were either untreated or „deoxygenated”by boiling for 15 min and then stored under a He atmosphere for three months. For all experiments the vials/bottles were placed on a small rocking table—gently shaking the vials/bottles for 15 min per hour, data points acquired during shaking are not included in the graphs because of electrical interference from the rocking table.

## Results and Discussion

### Configuration of the Readout Device

**Fig 1:**
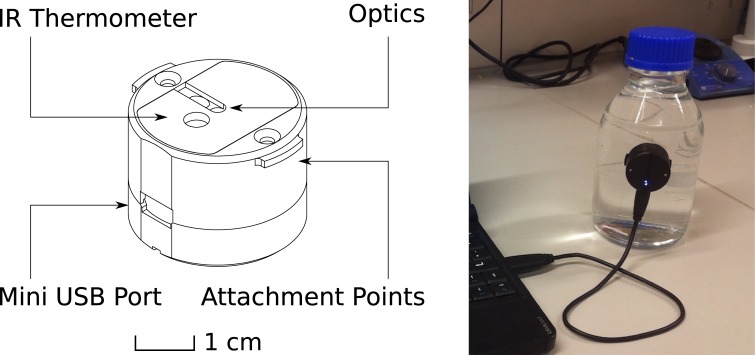
Device Configuration. A representation of the LUMOS readout device (left) and its application for measurements in a glass bottle (right). The LUMOS casing features small notches than can be used as attachment points for mechanical mounting of the device to a vessel wall. The flat front surface is also very well suited for using adhesive tape for mounting.

**Fig 2:**
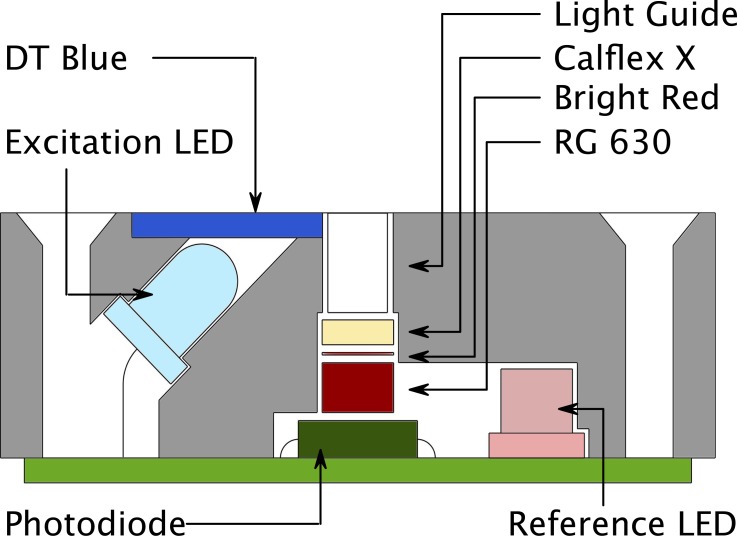
Optical Setup of the LUMOS. A cross section of the LUMOS‘ optical assembly. To maximize signal gain and simplify manufacturing a 45° angle layout is employed. The ideal distance of the sensor sheet from the top of the device is about 2–4 mm which is consistent with measurement through a vessel wall. Emission of the sensor is gathered in a short 3 mm fibre light guide and reaches the photodiode through a set of filters designed to reduce ambient and excitation light interference. The reference LED is positioned so that its light can directly reach the diode.

**Fig 3:**
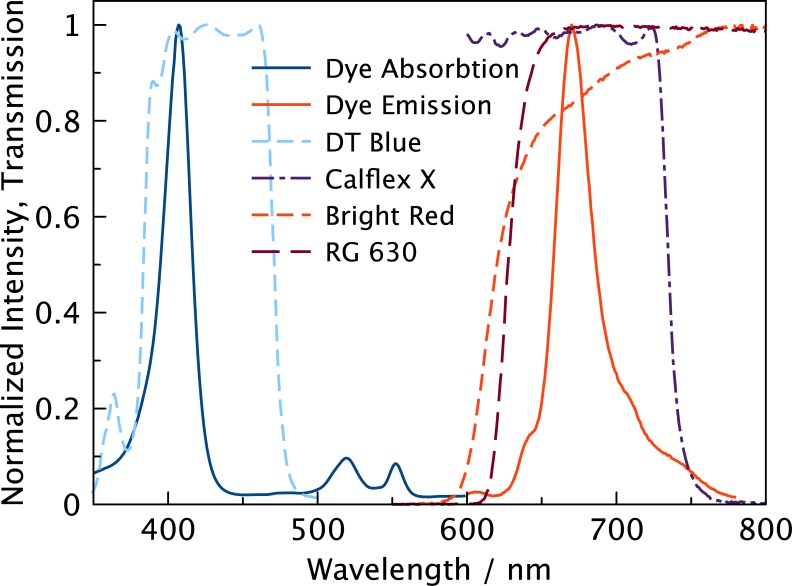
Spectral properties of the indicators and optical components. Transmission and normalized intensity spectra of the optical filters and the Pd-TFPP dye. A 405 nm LED is used to excite the dye and a DT Blue interference filter (the shown spectrum is the transmission at a 45° angle) is used to ensure that no excitation light is in the relevant detection region. A stack of Bright Red and RG 630 color filters combined with a Calflex X interference filter form a band pass for the interesting emission region. The Bright Red filter is used to minimize fluorescence inherent to the RG 630 color filter.

The LUMOS is a phase fluorimeter in a miniaturized package, designed to work with sensor sheets coated on glass substrates (Fig 1). The excitation circuit consists of a main 405 nm light emitting diode (LED) and a second 630 nm reference LED. Tests showed that both types of diodes have similar temperature dependence of their properties. Both LEDs are controlled by LED Driver Circuits that use operational amplifiers to convert the micro controllers digital to analog converter (DAC) output to a current between 0 and 94 mA. Operation amplifiers and the metal oxide semiconductor field-effect transistors (MOSFET) were chosen for low energy consumption and fast transition times, facilitating modulation of the excitation output in the kHz region. The optical components are enclosed in a machined anodized aluminum casing that optically isolates the different light pathways and acts as a support (Fig 2). The main LED is fixed at an angle which results in an optimal overlap of excitation and detector light pathways 2–4 mm outside of the device. In front of the LED a DT Blue shortpass interference filter assures that almost no light (< 0.1%) from the main LED can directly reach the photodetector circuit. Light that is emitted from the sensor material is collected in a cylindrical light fibre and guided through a Bright Red and RG 630 long-pass color filter combined with a short-pass Calflex X IR blocker to greatly reduce ambient light interference and background to under 0.5% (Fig 3). The emission light is then detected by a photodiode which is „bootstrapped“, resulting in reduced capacitance that improves the bandwidth of the amplifier, and a small reverse bias that reduces temperature dependence of the photodiode.

The current output of the photodiode is amplified using a 25 MOhm variable gain amplifier, and the resulting voltage is input to the micro controllers analog to digital converter (ADC). Because of the low modulation frequencies required for phosphorescence readout, all the signal processing can be done digitally and the need for an analog lock-in amplifier is eliminated, allowing for the small form factor of the device. Instead of a lock-in amplifier a digital single frequency Fourier transform algorithm is applied to the ADC input and in conjunction with coherent averaging the phase shift at the excitation frequency is then computed.

In a typical measurement the DAC outputs a sine wave represented by 64 points per period repeated over a sampling interval of 128 ms. This output is used to drive the main LED which in turn emits violet light that excites the dye in a sensing layer fixed in front of the device (usually inside a glass container, to which the readout device is attached on the outside, Fig 1, right). The luminescence response of the dye, which is phase shifted with respect to the excitation light, is guided to the photodiode, and the resulting current is amplified and read into the ADC as a voltage. Afterwards the reference LED is activated with the same DAC output, and because of the special geometry its emission light is directly guided to the photodiode. Any phase shift measured is thus solely due to electronic lag time. This reference value in conjunction with a previously performed zero calibration and the phase shift measured in the previous step are used to obtain the real phase shift value for the sensor material. This phase shift value can then be converted into a luminescence lifetime τ (assuming mono-exponential behavior of the decay curve) with the formula:

τ = tan(ɸ)/(2π f)

where f is the frequency of the modulation and ɸ is the phase shift. A calibration can then be applied for obtaining oxygen concentration values. For temperature compensation purposes an IR temperature sensor is also included in the package: it measures thermal emission in the μm wavelength range (where glass has an emissivity close to 1), and therefore allows for online temperature compensation. The obtained phase shift and temperature values are placed into registers and can be read out by any protocol conforming software on an attached PC. The communication is based on a virtual com port provided via a UART to USB interface chip by FTDI. This chip is contained on a printed circuit board separate to that of the measurement circuitry and also controls advanced behavior like providing a hard reset and JTAG Interface for debugging and interfacing with the micro controllers boot loader for firmware updates. This separate board also contains the power control circuit that supplies the measurement circuit with a stabilized voltage.

### Sensing Chemistry

Reaching detection limits in the low nanomolar region is not a trivial task. There are multiple factors that influence the performance of a sensor, some of them inherent to the dye used and some to the matrix in which the dye is usually immobilized in. Both of these components have to be optimized to create sensor materials for measuring trace oxygen concentrations. Generally the sensitivity of an optical sensing material has to be estimated, acknowledging that permeability of the matrix multiplied by luminescence lifetime is directly proportional to sensitivity.[19] Using highly permeable matrices and dyes with long luminescence decay times is therefore the most reliable way to reach high sensor sensitivity.

A variety of dyes with very long decay times exist, with porphyrin based dyes being very popular[20–23]. Especially benzoporpyrin based dyes are known for their high photostability and high molar absorption coefficients[22]. There are two viable choices of porphyrin dyes: 1) benzoporphyrins and 2) pentafluorophenyl substituted porphyrins.

Benzoporphyrins feature absorption maxima in the blue and red region of the electromagnetic spectrum and luminescence in the NIR region. The lifetimes of Pd (II) benzoporphyrin complexes are usually in the range of 200–400 μs, depending on substitution pattern and solubility in a given matrix. Because of their prominent absorption maximum in the red region they can be excited with ~600 nm LED light, which has some application specific advantages like higher transmission through tissue and lower levels of autofluorescence compared to excitation with UV light. Due to the NIR luminescence, visible light can be removed by filters and ambient light interference is therefore minimized. The dyes show very good solubility in standard matrices like polystyrene.

Pentaflourophenyl substituted porphyrins (S1 Fig) feature efficient absorption in the violet part of the spectrum and luminescence in the red region (Fig 3). Photostability of these dyes is known to be the highest among oxygen indicators. They have even longer lifetimes than most benzoporphyrins; up to 1200 μs in common organic solvents for the Pd(II) complex. They are also soluble in most standard matrices, but in addition, their perfluorinated substituents render them soluble in highly permeable perfluorinated polymers like Hyflon (S1 Fig) and Teflon AF.

A sensor system could have been realized with both types of dyes, but the LUMOS ultimately was designed to be compatible with the pentafluorphenyl porphyrins Pd-TFPP and its spectrally comparable platinum(II) complex Pt-TFPP. The higher susceptibility to ambient light interference was considered a justifiable trade-off considering the expected 2-fold increase in sensitivity due to longer luminescence decay times alone. Also Pd-TFPP and Pt-TFPP are available commercially and can be embedded in the required high permeability matrices without further chemical modification.

Regarding the matrix for the sensing material, the choices were rather limited. Polystyrene is often used as a standard material, and combined with Pd-TFPP it would feature adequate sensitivity (although 10x lower than the chosen material, S2a Fig), with a detection limit already in the nM region. However, due to oxygen consumption in the sensor film itself, which is caused by the matrix not being stable enough against the very reactive singlet oxygen species generated during quenching, polystyrene could is less suitable for the sensor[24]. Other possible materials include ormosils, which have been shown to feature higher oxygen permeabilities[25,26]. Indeed using poly(phenylsilsesquioxane) (PPSQ) as material increased the resulting sensors sensitivity 4-fold (S2b Fig). However, adsorption onto silica gel surface promised even higher sensitivities, and while this approach requires covalent grafting of the dye onto the surface, the resulting sensors are still the most sensitive based on that specific dye known to us[27]. Unfortunately there are some drawbacks of this approach, like low batch-to-batch reproducibility of sensor properties, more complex sensor preparation and lower long-term stability, due to chemical degradation of the more accessible dye. As a result, perfluorinated matrices such as Hyflon AD 60 (P ≈ 170 × 10^–16^ mol^-1^ m^-1^ s^-1^ Pa^-1^, ref. [28]) and Teflon AF 1600 (P ≈ 1200 × 10^–16^ mol^-1^ m^-1^ s^-1^ Pa^-1^, ref. [29]) were chosen, as they feature excellent chemical stability and are also commercially available. While sensors based on Teflon AF could potentially feature sensitivities comparable to silica-gel based sensors, it was concluded that the much cheaper Hyflon AD 60, in which the dyes also show better solubility and lower temperature dependence, should be used as the matrix material.

The final sensor materials thus consisted of Pd-TFPP embedded in Hyflon AD 60 (trace range, S3 Fig), and Pt-TFPP embedded in Hyflon AD 60 (high range, S4 Fig). These two sensors can be used to achieve an overall higher dynamic range and both are compatible with the LUMOS readout device by just changing the excitation frequency (171 Hz for Pd-TFPP, 1500 Hz for Pt-TFPP). Both sensors feature very low temperature crosstalk in τ_0_ and negligible temperature crosstalk in K_SV_ (S3 and S4 Figs). Their calibration curves are not linear, but can be fitted using the two-site Stern-Volmer model[30,31]. K_SV_ is about 6.25 x 10^–3^ ppmv^-1^ for the trace material and 0.29 x 10^–3^ ppmv^-1^ for the high range material, which corresponds to detection limits of 0.5 nM and 10 nM, respectively (assuming a minimal detectable phase shift change of 0.05°). The upper range limits are approximately 1000 nM and 20 μM for the trace and high range sensing material, respectively (assuming a cut-off at τ_0_/ τ ≈ 4). Both sensors are usable in even higher ranges but noise levels will increase noticeably. The low detection limits combined with low noise and high sampling frequency make the LUMOS ideally suited for monitoring rates and oxygen levels in the low nano molar region.

### Applications

**Fig 4:**
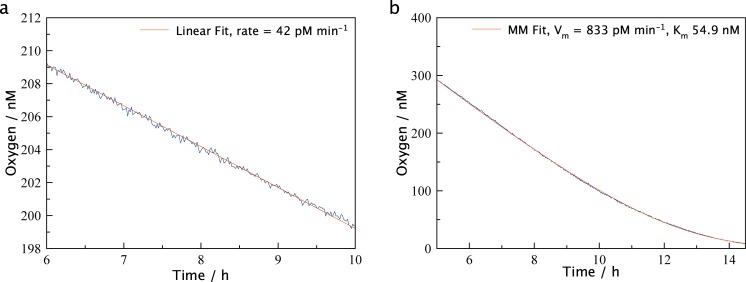
Respiration Experiments. Selected regions of a microbial respiration in tap-water (a) and tap-water with added manure (b). The more active sample shows considerably higher consumption rates and in both samples the low noise of the LUMOS is evident. In (a) a linear fit (red line) is applied, but in (b) with manure amended water a Michealis-Menten fit (red line) can be applied due to the larger concentration range being analyzed.

The device was developed as a tool for marine biologists and as such has been employed in a number of laboratory and field experiments. Presentation of the variety of data obtained with the LUMOS is outside the scope of this manuscript and will be published separately. Here we include some sample results as a demonstration of sensor performance during some representative applications. Fig 4 shows measurements in tap-water before and after amendment with diluted cattle manure (Fig 4a and 4b). Prior to manure addition the oxygen decline was linear reflecting a consumption rate of 42 pmol L^-1^ min^-1^. The microbial activity was greatly stimulated after manure addition and the acquisition of a larger part of the curve allowed for direct fitting of an integrated Michaelis Menten kinetic[32], resulting in a V_m_ of 833 pmol L^-1^ min^-1^ and a K_m_ of 54.9 nM. Noise was below one nanomolar.

**Fig 5:**
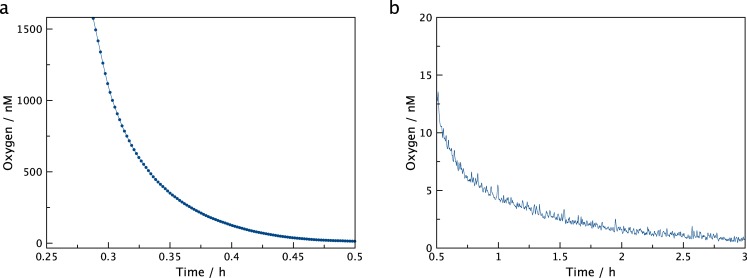
Enzymatic oxygen consumption. Oxygen consumption in buffered glucose solution catalyzed by glucose oxidase showing the fast initial consumption (a) and slower consumption at low nM concentrations (b).

Another example of oxygen consumption in the trace oxygen region is depicted in Fig 5. Here the oxygen consumption of glucose oxidation catalyzed by glucose oxidase was monitored. The data quality is similar to the example above, and demonstrate fast low-noise determination of oxygen concentrations over three orders of magnitude. Michaelis Menten fits did, however, result in poor correlation coefficients, presumably as glucose was not available in excess and no catalase was present.

**Fig 6:**
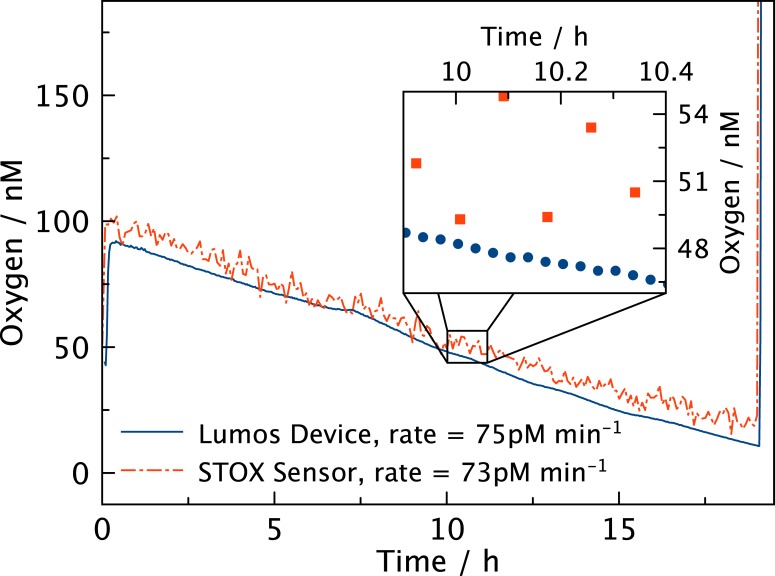
Comparison of the LUMOS and STOX sensor. Respiration in a deep sea sample monitored with both LUMOS and a STOX electrochemical sensor simultaneously. Both sensors detect comparable rates but the much lower noise of the LUMOS is evident.

Electrochemical STOX sensors have previously been applied in aquatic studies of trace oxygen dynamics[13]. Fig 6 presents the results of a parallel measurement of respiration rates using LUMOS and a STOX sensor in a sea water sample from near anoxic OMZ deep water. Both devices show comparable rates and parallel trends and the slight parallel shift might be a result of differences in calibration procedures. However, the better signal to noise ratio of the LUMOS is evident. Even though neither of the devices operate at their maximum sampling frequencies the LUMOS has a distinct advantage in supplying a virtually continuous oxygen reading whereas the STOX sensor only yields one value for every measuring cycle that typically takes 0.5–5 min. A measuring cycle of a STOX sensor consists of a period where oxygen is allowed to enter the sensor followed by a period where oxygen is prevented from reaching the sensing cathode, representing the control of zero signal. This switching results in the somewhat lower response time, but is also the feature that allows for comparatively better resolution at low concentrations as compared to other electrochemical oxygen sensors[18].

**Fig 7:**
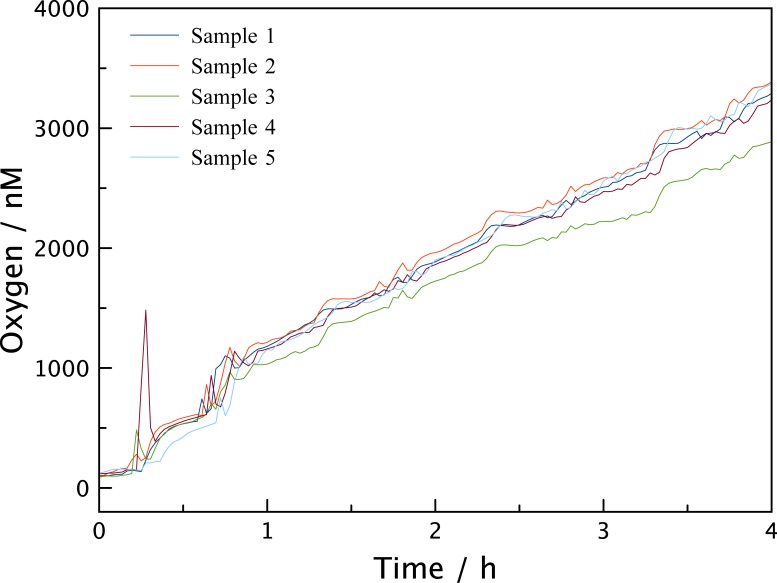
Oxygen Level in Exetainer vials. Measurement of the oxygen intrusion into 12 ml Exetainer vials that were used without special preparations to reduce oxygen contamination. The initial low oxygen concentration quickly rises to above 1 μM in less than an hour. The five lines represent five simultaneous measurements in separate vessels.

**Fig 8:**
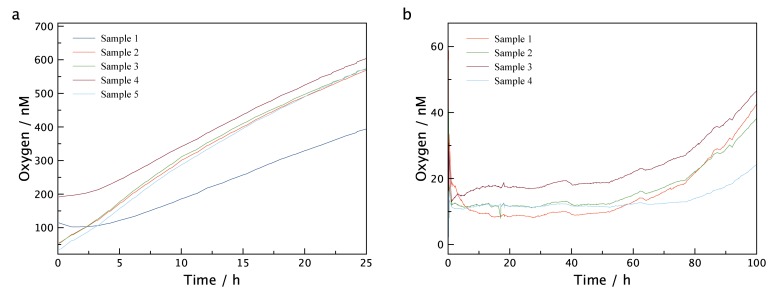
Oxygen Level in Exetainer vials with oxygen contamination reducing preparations. Measurement of the oxygen contamination in 12 ml Exetainer vials using rubber seals that were stored under helium atmosphere for one month (a) and additional introduction of a 2 ml He headspace (b). Both experiments show significantly reduced oxygen ingress.

Due to the capability of the LUMOS to measure through transparent walls of an experimental vessel, the device could be used to assess the ingress of oxygen into the type of containers that are routinely used to experimentally incubate samples from low oxygen environments. The results of these measurements can be seen in Fig 7 where a glass vial with a butyl rubber septum (Exetainer) was filled with degassed water amended with HgCl_2_ to inhibit any microbial activity. Vials were placed at constant temperature and exposed to air. No special precautions like the ones described by De Brabandere et al.[33] were taken to avoid oxygen contamination and multiple LUMOS setups were applied in parallel. After just one hour, oxygen levels within the vials had increased to approximately 1 μM, and after 4 h the concentrations were around 3 μM. As such vials are routinely used for measuring anaerobic metabolism the 3 μM contaminating oxygen is worrisome as this would be sufficient oxygen to sustain aerobic metabolism and possibly inhibit anaerobic metabolism. For the water samples presented in Fig 6, 3 μM corresponds to 30 days of respiration. The experiment was subsequently repeated using: i) caps that had been stored under helium atmosphere for one month and ii) an additional 2ml headspace of He introduced into the vials. Fig 8 shows that both experiments exhibit drastically reduced oxygen contamination rates, suggesting that the main reason for contamination is diffusion from the oxygen contained in the rubber seal. Especially the vials containing the additional headspace show a constant low level of oxygen for up to 50 hours, making this method of preparation the preferred method to store samples with very low oxygen content. In S5 and S6 Figs the experiments were repeated with 120 ml serum bottles, and although overall oxygen contamination was lower due to the higher volume, the same general trend can be observed and the contamination was still significant.

It should be mentioned here that although the “sensing chemistry” was optimized for detection of very low concentrations of oxygen, the LUMOS fluorimeter can be used in combination with any other spectrally compatible sensing material. For example, all sensors based on Pt(II) and Pd(II) pentafluorophenyl porphyrin and octaethylporphyrin can be read-out with LUMOS; the sensors based on NIR-emitting benzoporphyrin complexes can be also interrogated if the optical emission filters are exchanged to match the spectral properties of these indicators. Thus, the planar sensor spots and water-dispersible nanoparticles designed for measurements in physiologically relevant range (up to 250 μmol L^-1^ O_2_) can be also applied. This enables numerous applications in biology, medicine and biotechnology.

## Conclusions

We developed a combined system of specialized sensing materials and an optimised readout device that allows for high resolution oxygen sensing in the 0.5–1000 nM range. This set up is ideal for studying respiration rates and microbial activity at trace amounts of oxygen. A second sensor material that is also compatible with the readout device can be used for higher concentration regions of 10–20.000 nM. As a result the whole system is small, modular, flexible, robust and easy to use, making it a valuable tool for researchers.

## Supporting Information

S1 Fig Chemical structures of the Indicators Pt-TFPP and Pd-TFPP and Hyflon AD. (EPS)

S2 Fig Calibration in standard matrices. Calibrations of Pd-TFPP in polystyrene (a) and PPSQ (b) at 20°C.
(EPS)

S3 Fig Calibration of the Pd-TFPP-based trace sensor. Calibration of the LUMOS trace sensor material (Pd-TFPP in Hyflon AD 60) at different temperatures. Notice the high sensitivity and low temperature crosstalk in τ_0_(a), as well as the negligible temperature crosstalk in K_SV_(b). Calibration curves show a slight curvature, that is common for polymer based matrices and can be fitted using the two-site Stern-Volmer model.
(EPS)

S4 Fig Calibration of the Pt-TFPP-based trace sensor. Calibration of the LUMOS high range sensor material (Pt-TFPP in Hyflon AD 60) at different temperatures. This material also shows very low dependencies of τ_0_ (a) and K_SV_ (b) on temperature. The sensitivity of this material is around twenty fold smaller than the trace material and provides a larger dynamic range for measurements at higher oxygen concentrations.
(EPS)

S5 Fig Oxygen Level in Serum Bottle. Measurement of the oxygen intrusion into samples contained in three 120 ml serum bottles that were used without special preparations to reduce oxygen contamination. While oxygen intrusion is smaller compared to Exetainer vials, in part due to the larger volume to exposed surface ratio, intrusion is still significant.
(EPS)

S6 Fig Oxygen Level in Serum Bottle with oxygen contamination reducing preparations. Measurement of oxygen intrusion into samples contained in 120 ml serum bottles capped with stoppers that were kept under helium atmosphere for 3 months (a) and an additional 20 ml He headspace (b). In the same trend as Fig 8, oxygen intrusion is vastly reduced especially for the bottles with headspace.
(EPS)
